# The genus *Ussurohelcon* Belokobylskij (Hymenoptera, Braconidae, Helconinae) in Vietnam, with descriptions of six new species

**DOI:** 10.3897/zookeys.1266.176930

**Published:** 2026-01-14

**Authors:** Nguyen Thi Oanh, Nguyen Van Dzuong, Khuat Dang Long, Cornelis van Achterberg, Nguyen Duc Hiep

**Affiliations:** 1 Dong Thap University, Pham Huu Lau Road, Cao Lanh, Dong Thap, Vietnam Dong Thap University Dong Thap Vietnam; 2 Tay Bac University, Son La, Vietnam Tay Bac University Son La Vietnam; 3 Institute of Biology, Vietnam Academy of Science & Technology, 18 Hoang Quoc Viet Road, Cau Giay, Ha Noi, Vietnam Institute of Biology, Vietnam Academy of Science & Technology Ha Noi Vietnam; 4 Naturalis Biodiversity Center, Postbus 9517, 2300 RA Leiden, Netherlands Naturalis Biodiversity Center Leiden Netherlands

**Keywords:** Cenocoeliinae, Helconinae, Ichneumonoidea, new record, Oriental, parasitoids, Ussurohelconini

## Abstract

The genus *Ussurohelcon* Belokobylskij is recorded for the first time from Vietnam with six new species described and fully illustrated: *Ussurohelcon hatinh* Long, **sp. nov**., *U. hagiang* Long, **sp. nov**., *U. mellicentralis* Long, **sp. nov**., *U. mocchau* Long & van Achterberg, **sp. nov**., *U. similis* Long, **sp. nov**. and *U. tuyenquang* Long, **sp. nov**. Comparative morphological characters of *Ussurohelcon* species are discussed and a key to Oriental species is also provided.

## Introduction

The genus *Ussurohelcon* Belokobylskij, 1989 is a small, mainly East Asian genus originally included in the Helconinae (Belokobylskij 1989). Later this genus was transferred to Cenocoeliinae and placed in Ussurohelconini by van Achterberg (1994) (van Achterberg 1994; Belokobylskij 1998). However, according to Sharanowski et al. (2011), the Ussurohelconini belong to the subfamily Helconinae. Until now, *Ussurohelcon* has consisted of five species, of which three species are from the Oriental region, one is from the Australian (Wallacea) region, and one is from the Eastern Palaearctic region (Yu et al. 2016).

In Vietnam, four genera of Helconinae have been reported in scattered papers (Long and Belokobylskij 2003; Long and van Achterberg 2014; Long et al. 2020), of which no *Ussurohelcon* species had been reported. In this study, six species belong to *Ussurohelcon* are described as new for science. *Ussurohelcon* is also the first recorded for Vietnam’s braconid fauna. Biology of *Ussurohelcon* is unknown; however, by including this genus in the subfamily Helconinae, *Ussurohelcon* species are likely also endoparasitoids of larvae of wood- and bark-boring Coleoptera. Of the six *Ussurohelcon* species reported herein from Vietnam, a series of 13 females of the new species *U. mocchau* sp. nov., emerged from wood fallen from a dead tree in a semi-open habitat (Fig. 10).

## Materials and methods

Materials examined came from the Hymenopteran Collection, the Institute of Biology (formerly Institute of Ecology and Biological Resources, IEBR), Vietnam Academy of Science and Technology (**VAST**) Ha Noi Vietnam. Portions of examined specimens were mainly collected with funding from the National Science Foundation Grant DEB-9870232: “Collaborative research: Multi-taxa inventory of threatened conservation areas in Vietnam” 1998–2003. All types of the new species are deposited in the Institute of Biology, except for two paratypes of *Ussurohelcon mocchau* Long & van Achterberg, sp. nov., which deposited in the American Museum of Natural History, New York (**AMNH**), and the Naturalis Biodiversity Centre, Leiden, the Netherlands (**RMNH**).

Vein terminology follows the modified Comstock–Needham system terminology, and sculpture terms are based on Harris (1979) and van Achterberg (1993). The diagnosis of the genus is conducted according to Belokobylskij (1989). Besides, the diagnosis of the recognition of the tribe Ussurohelconini is based on van Achterberg (1994). Moreover, a diagnosis of the tribe is carried out as described by van Achterberg (1994). In addition, additional references and data follow Belokobylskij (1989), van Achterberg (1994), and Yu et al. (2016).

We used an Olympus^®^ SZ61 binocular microscope. The photographs were made with a Sony^®^α7R digital camera attached to a Nikon^®^ SMZ800N binocular microscope and Helicon Focus^®^ 8 stacking software. The morphological features were slightly processed with Adobe Photoshop CS5 to adjust their size and background. The figures were intended to be used comparatively, which help greatly in progressing through the key. The scale bars in the figures indicate millimetres.

Abbreviations used in this paper are as follows:

“Hel.+**number**” Code number indexing Helconinae specimens in the Hymenoptera collection at IEBR

**OD** Diameter of posterior ocellus

**OOL** Minimum ocular–ocellar distance

**POL** Minimum postocellar line

## Results

### Family Braconidae Nees, 1811

**Subfamily Helconinae Förster, 1863**

**Tribe Ussurohelconini van Achterberg, 1994**

#### Genus Ussurohelcon

Taxon classificationAnimaliaHymenopteraBraconidae

Belokobylskij, 1989

E53D30D7-C78A-523F-A156-26D064C7D3A0

Ussurohelcon Belokobylskij, 1989: 25. Type species (by original designation): Ussurohelcon longigenis Belokobylskij, 1989—van Achterberg 1994: 6; Yu et al. 2016.

##### Diagnosis.

*Ussurohelcon* is distinguishable from other genera of Helconinae by a combination of the following characters: occipital carina strongly bent towards and ventrally joined to hypostomal carina; head elongate; propleuron flattened; postpectal carina absent in front of middle coxa, present only at end of mesosternal sulcus; propodeal spiracle near middle of propodeum; vein 1-SR of fore wing absent or present but very short; vein 2A of hind wing present, strongly inclivous; marginal cell of hind wing somewhat widened distally; metasoma virtually smooth; ovipositor sheath longer metasoma.

### Checklist and distribution of *Ussurohelcon* species

*Ussurohelcon annulicornis* van Achterberg, 1994. Oriental: Malaysia (Sabah)

*Ussurohelcon celebensis* van Achterberg, 1994. Australian: Indonesia (Sulawesi)

*Ussurohelcon hagiang* Long, sp. nov. Oriental: Vietnam

*Ussurohelcon hatinh* Long, sp. nov. Oriental: Vietnam

*Ussurohelcon koshunensis* (Watanabe, 1934). Oriental: China (Taiwan)

*Ussurohelcon longigenis* Belokobylskij, 1989. Eastern Palaearctic: Russia (Primorskiye Kray)

*Ussurohelcon mellicentralis* Long, sp. nov. Oriental: Vietnam

*Ussurohelcon mocchau* Long & van Achterberg, sp. nov. Oriental: Vietnam

*Ussurohelcon nigricornis* van Achterberg, 1994. Oriental: Malaysia (Sabah)

*Ussurohelcon similis* Long, sp. nov. Oriental: Vietnam

*Ussurohelcon tuyenquang* Long, sp. nov. Oriental: Vietnam

### Key to Vietnamese *Ussurohelcon* species

**Table:** 

1	Frons rugo-punctate (Fig. 12A); vein r-m of fore wing vertical (Fig. 12H)	***Ussurohelcon similis* Long, sp. nov**.
–	Frons discretely punctate entirely or at least laterally (Figs 2A, 4A, 6A, 9A); vein r-m of fore wing virtually inclivous (Figs 4H, 6F, 7I, 9I, 14I) [vertical in *Ussurohelcon hagiang* sp. nov (Fig. 2I)]	**2**
2	Frons with rather shallow median depression; vein r-m vertical or nearly so (Fig. 2I)	***Ussurohelcon hagiang* Long, sp. nov**.
–	Frons with rather deep median depression; vein r-m inclivous (Figs 4H, 6F, 9I, 14I)	**3**
3	Vein 1-SR of fore wing absent (Fig. 9I); vein 1-M weakly curved; face with median longitudinal groove from frons to clypeus (Fig. 9B); precoxal sulcus shallowly impressed throughout, rugulose (Fig. 9E)	***Ussurohelcon mocchau* Long & van Achterberg, sp. nov**.
–	Vein 1-SR of fore wing present but short (Figs 4H, 6F, 14I); vein 1-M distinctly curved basally or throughout (Figs 4H, 6F, 14I); face without median longitudinal groove (Figs 4B, 6B, 14B); precoxal sulcus deeply impressed anteriorly, smooth or coriaceous (Figs 4E, 6E, 14E)	**4**
4	Propleuron black (Fig. 14E); mesonotum black (Fig. 14D); first metasomal tergite slender, 1.5× as long as its posterior width (Fig. 14H); vein 1-CU1 of fore wing 0.7× as long as cu-a (Fig. 14I); vein SR1 of fore wing distinctly curved subapically; veins 1A of fore wing developed, nearly sclerotized (Fig. 14I); ovipositor sheath 1.5× fore wing	***Ussurohelcon tuyenquang* Long, sp. nov**.
–	Propleuron yellow or pale brown (Figs 4E, 6E); mesonotum entirely yellow or yellowish brown (Figs 4D, 6D); first metasomal tergite robust, 1.2–1.3× as long as its posterior width (Figs 4J, 6G); vein 1-CU1 of fore wing as long as cu-a (Figs 4H, 6F); vein SR1 of fore wing straight or weakly sinuate; vein 1A unsclerotized (Figs 4H, 6F); ovipositor sheath 1.0–1.3× fore wing	**5**
5	Mesonotum yellowish brown (Fig. 4D); occipital carina complete, evenly curved medio-dorsally; stemmaticum rugose (Fig. 4A); vein 1-M of fore wing distinctly only curved basally (Fig. 4H); vein 3-SR of fore wing 0.8× as long as 2-SR; ovipositor sheath 1.3× fore wing	***Ussurohelcon hatinh* Long, sp. nov**.
–	Mesonotum yellow (Fig. 6D); occipital carina angularly interrupted medio-dorsally; stemmaticum finely punctate (Fig. 6A); vein 1-M of fore wing distinctly curved medially (Fig. 6F); vein 3-SR of fore wing as long as 2-SR; ovipositor sheath as long as fore wing	***Ussurohelcon mellicentralis* Long, sp. nov**.

### Description of species

#### Ussurohelcon hagiang

Taxon classificationAnimaliaHymenopteraBraconidae

Long sp. nov.

7AFD8929-A060-5B6D-AEC4-E52A0546D7E6

https://zoobank.org/FC5426B9-429D-4C83-8A06-27521E5E5BBE

Figs 1, 2

##### Type material.

***Holotype***: • ♂, “Hel.**121**” (IEBR), Northeast Vietnam: Ha Giang, Vi Xuyen, Cao Bo, Tay Con Linh Mountains, forest, 20°47'N, 104°49'E, 1400 m, sweep (net),14.ix.2000, K.D. Long.

**Figure 1.**
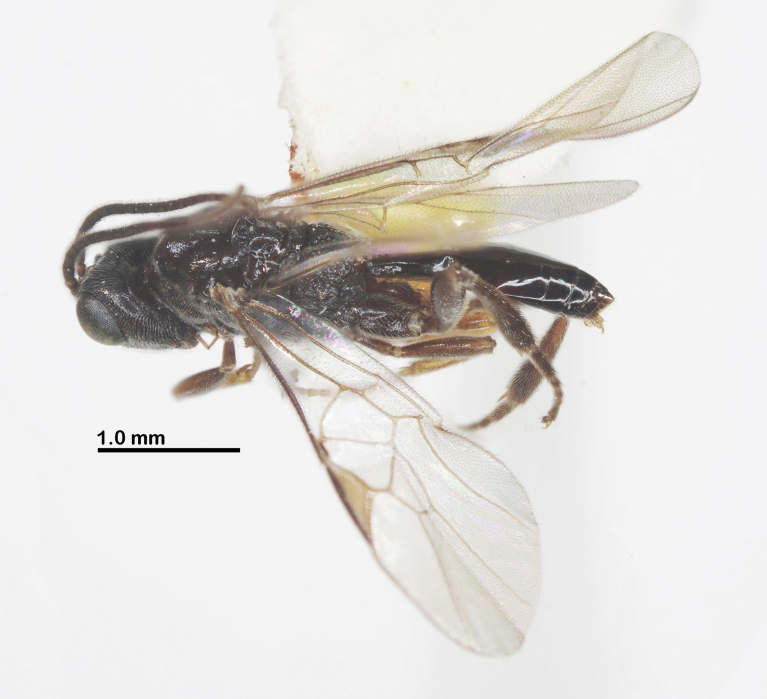
Habitus of *Ussurohelcon hagiang* Long, sp. nov., holotype, male, dorso-lateral view.

**Figure 2.**
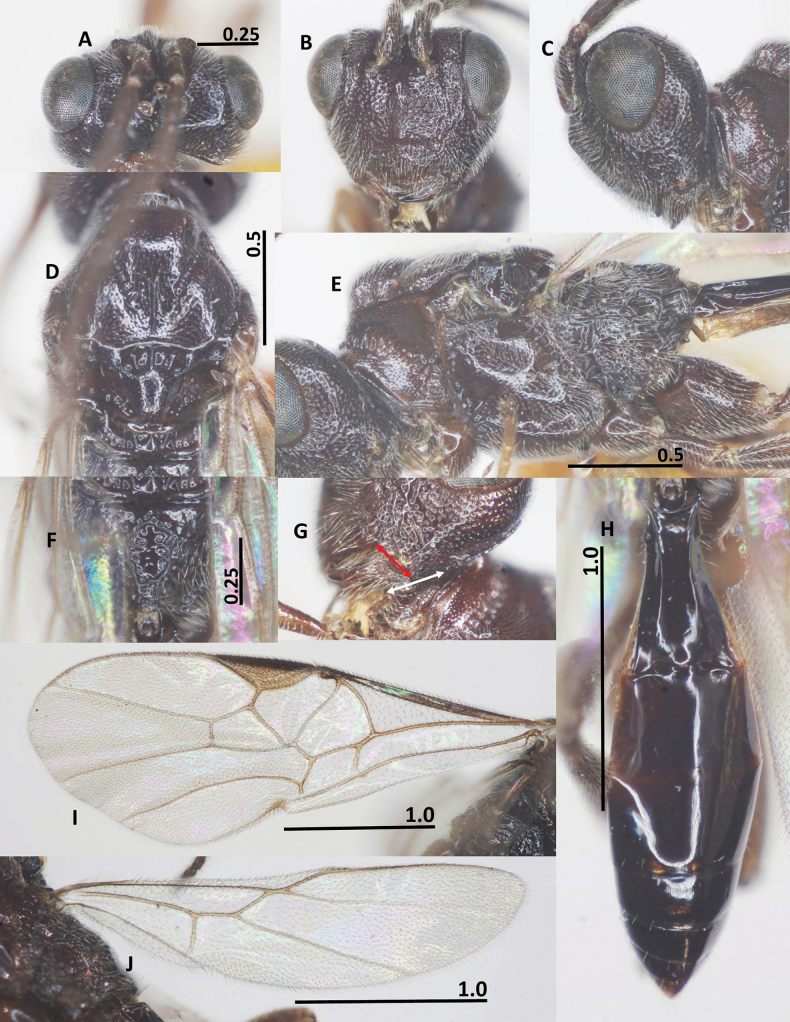
Diagnostic characters of *Ussurohelcon hagiang* Long, sp. nov., holotype, male. **A**. Head, dorsal view; **B**. Head, frontal view; **C**. Head, lateral view; **D**. Mesonotum, dorsal view; **E**. Mesopleuron; **F**. Propodeum; **G**. Occipital flange and mandible, red arrow indicates width of mandible, and white arrow indicates length of ventral occipital flange; **H**. Metasoma, dorsal view; **I**. Fore wing; **J**. Hind wing. Scale bars in mm.

##### Notes.

*Ussurohelcon hagiang* sp. nov. is the most similar to *U. longigenis* Belokobylskij, 1989 from the Eastern Palaearctic (Russia), but it differs from the latter in having the propodeum with a closed areola (vs open in *U. longigenis*), the frons largely punctate laterally (vs finely punctate in *U. longigenis*), and OOL 2.5× OD (vs 4.0× in *U. longigenis*).

##### Description.

Holotype, ♂, length of body 3.6 mm, fore wing 3.3 mm (Fig. 1).

***Head***. Antenna with 27 flagellomeres; antenna without white band; length of scapus 3.0× its maximum width; length of first flagellomere 1.1× second one; length of first, second, and penultimate flagellomeres 2.5, 2.3, and 1.5× their widths, respectively; in dorsal view, head 2.0× as wide as length medially; eye 2.0× as long as temple (Fig. 2A); frons depressed, medially smooth, laterally punctate; lamella between antennal sockets protruding above depression, anteriorly connecting ridge; occipital carina dorsally complete, medio-dorsally mostly curved; ocelli small, OOL: OD: POL = 10: 4: 5; vertex and temple finely punctate; in frontal view, width of face 0.7× length of face and clypeus medially combined; eye 1.4× as long as malar space; face with obtuse median ridge, densely rugo-punctate (Fig. 2B); clypeus not medially depressed, rugo-punctate, with indistinct pointed medio-ventral process; occipital flange not overpassing 0.3 basal of mandible, 1.2× as long as basal width of mandible (Fig. 2C, G); in lateral view, eye length 1.4× as long as its width, and 2.1× malar space, respectively; malar space 2.5× as long as basal width of mandible; malar space largely rugulose; mandible rugo-punctate, smooth at extreme apex (Fig. 2G); length of maxillary palp 0.5× height of head.

***Mesosoma***. In lateral view, mesoscutum flat posteriorly, length of mesosoma 1.55× its height (Fig. 2E); pronotal side densely crenulate medially, finely punctulate dorsally and ventrally; propleuron narrow, finely punctulate (Fig. 2E); prepectal carina complete, strong; postpectal carina present only medio-posteriorly; precoxal sulcus wide, shallowly impressed, but deeper near postpectal carina, sparsely punctate, with dense long setae posteriorly; remainder of mesopleuron (dorsally and ventrally) finely punctulate (Fig. 2E); metapleuron with long setae, largely rugo-punctate; notauli narrow, sparsely crenulate anteriorly, fused into flat area posteriorly; median lobe of mesoscutum finely punctulate; lateral lobes convex rugo-punctate anteriorly, coriaceous posteriorly; medio-posterior carina on 0.5 apical of the median lobe of mesoscutum (Fig. 2D); scutellar sulcus deep and wide, 0.5× as long as scutellum, with three carinae; scutellum convex and finely punctulate, propodeum with short basal carina and closed median areola, reticulate rugose laterally, smooth medially (Fig. 2F).

***Wings***. Fore wing: pterostigma 3.3× as long as its width medially (Fig. 2I); r: 3-SR: SR1 = 5: 11: 46; 2-SR: 3-SR: r-m = 14: 11: 6; 3-SR: 2-M = 11: 18; vein r-m vertical or nearly so (Fig. 2I); vein m-cu far postfurcal; 1-CU1: cu-a: 2-CU1: 3-CU1 = 5: 8: 13: 7; vein 1-SR present and short; vein 1-M distinctly curved; vein a absent, vein 2A weakly indicated at base. Hind wing: M+CU: 1-M: cu-a: 1r-m = 28: 9: 10: 8; 1-M: 1r-m = 9: 8 (Fig. 2I); vein cu-a nearly straight, weakly curved apically, area basad of it sparsely setose (Fig. 2J).

***Legs***. Length of fore tarsus 0.7× fore tibia; length of femur, tibia and basitarsus of hind leg 3.3, 6.6 and 5.4× their width, respectively; length of inner and outer hind tibial spurs 0.3 and 0.25× hind basitarsus, respectively.

***Metasoma***. Length of first metasomal tergite 1.7× its posterior width, its surface largely smooth (Fig. 2H), and dorsal carinae strong basally, extending up to 0.6 basal of the tergite; second suture indistinct.

***Colour***. Body blackish brown; palpi brown; glossa yellow; fore and middle legs pale brown, except fore and middle tibia and tarsus yellow; hind leg brown; tegula yellow; veins on wings yellowish brown, wing membrane hyaline; mesosoma chocolate brown; metasoma brown to dark brown.

**Female**. Unknown.

##### Etymology.

The new species is named after the type locality (Ha Giang province), Northeastern Vietnam.

##### Biology.

Unknown.

##### Distribution.

Northeastern Vietnam: Ha Giang province.

#### Ussurohelcon hatinh

Taxon classificationAnimaliaHymenopteraBraconidae

Long sp. nov.

3BE609A6-1E55-54D8-A0FD-F22664C32B5A

https://zoobank.org/C64F8498-6D78-4B77-B3AA-48B60A399AED

Figs 3, 4

##### Type material.

***Holotype***: • ♀, labelled “Hel.**073**” (IEBR), North-Central Vietnam: Ha Tinh, Huong Son, forest, 18°22'N, 106°13'E, 900 m, May 18, 1998, Malaise [trap], AMNH, K. Long.

##### Notes.

*Ussurohelcon hatinh* sp. nov. shares with *U. mellicentralis* sp. nov. frons discretely punctate, and with median depression rather deep; vein 1-SR of fore wing present, short; and vein r-m inclivous, but can be separated from the latter by the following characters: 1) Mesonotum yellowish brown (vs yellow in *U. mellicentralis*); 2) Vein 1-M of fore wing distinctly curved basally (vs distinctly curved medially in *U. mellicentralis*); ovipositor sheath 1.3× as long as fore wing (vs 1.0 × in *U. mellicentralis*). Differences between *U. hatinh* sp. nov. and *U. mocchau* sp. nov. indicated in the key.

**Figure 3.**
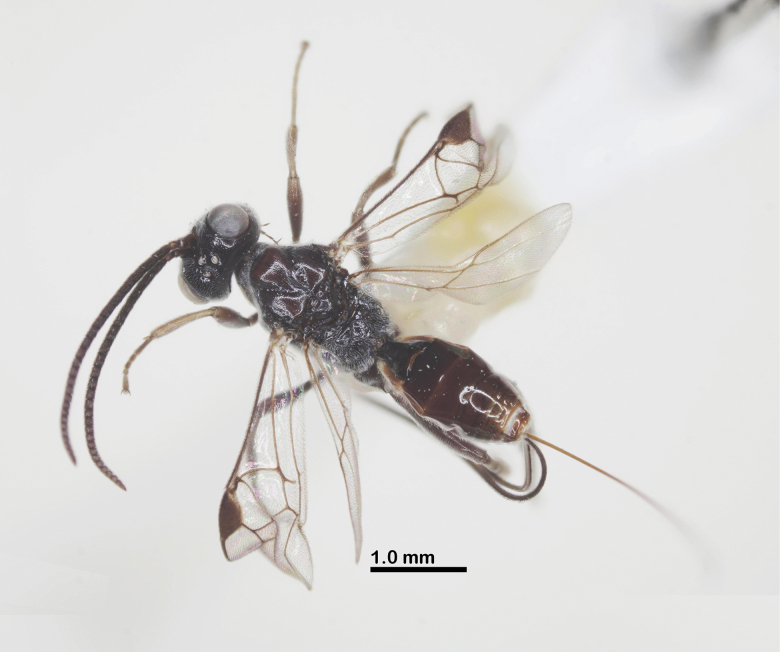
Habitus of *Ussurohelcon hatinh* Long, sp. nov., holotype, female, dorsal view.

**Figure 4.**
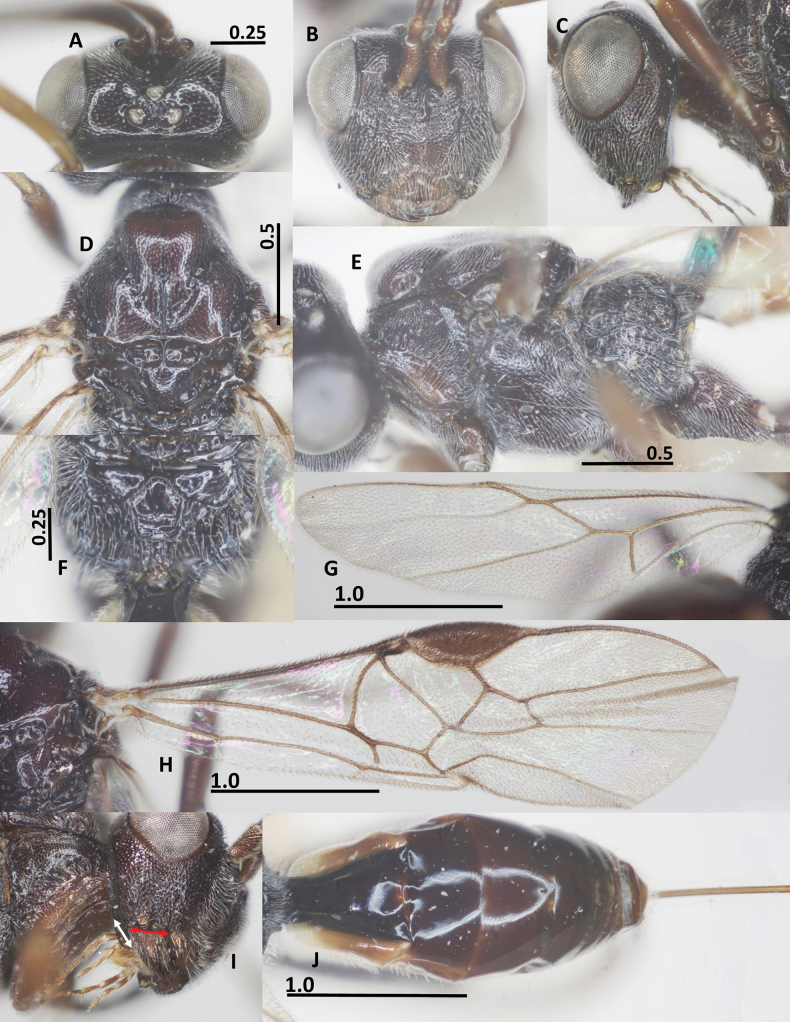
*Ussurohelcon hatinh* Long, sp. nov., holotype, female. **A**. Head, dorsal view; **B**. Head, frontal view; **C**. Head, lateral view; **D**. Mesonotum, dorsal view; **E**. Mesosoma, lateral view; **F**. Propodeum; **G**. Hind wing; **H**. Fore wing; **I**. Occipital flange and mandible, red arrow indicates width of mandible, and white arrow indicates length of ventral occipital flange; **J**. Metasoma, dorsal view. Scale bars in mm.

##### Description.

Holotype, ♀, length of body 4.3 mm, fore wing 3.5 mm, ovipositor sheath 4.4 mm (Fig. 3).

***Head***. Antenna with 24 flagellomeres; antenna without white band; length of scapus 3.0 × its maximum width; length of first flagellomere 1.2× second one; length of first, second and penultimate flagellomeres 3.25, 2.75, and 1.0× their widths, respectively; length of maxillary palp 0.5× height of head; in dorsal view, head 1.9× as wide as length medially; length of eye 2.5× temple (Fig. 4A); in dorsal view, occipital carina complete, medio-dorsally angulated; ocelli small, in high triangle; OOL: OD: POL = 11: 4: 5 (Fig. 4A); frons weakly anteriorly depressed, medially smooth, laterally sparsely punctate; vertex and temple sparsely punctate; in frontal view, lamella blunt, below level of antennal sockets, connecting obtuse median ridge (Fig. 4B); width of face 1.1× length of face and clypeus medially combined; distance between tentorial pits 0.7 × distance from pit to eye margin; eye length 1.4× malar space; face and malar space largely rugo-punctate; (Fig. 4B); clypeus depressed, rugo-punctate; ventral margin of clypeus with distinct pointed medial process; occipital flange protruding behind mandible, 1.0× as long as basal width of mandible (Fig. 4I); in lateral view, eye length 1.3–1.4× as long as its width and malar space; malar space 2.0× as long as basal width of mandible; malar space rugo-punctate; mandible twisted, basally finely rugo-punctate, apically smooth, ventrally with sparse setae (Fig. 4I).

***Mesosoma***. In lateral view, mesoscutum slightly raised posteriorly; length of mesosoma 1.4× its height (Fig. 4E); pronotal side largely medially crenulate, ventrally coriaceous, dorsally finely punctate; propleuron mostly coriaceous (Fig. 4E); prepectal carina complete, strong; postpectal carina curved, present only medio-posteriorly; precoxal sulcus shallowly impressed, but deeper near postpectal carina, nearly smooth; mesopleuron with small smooth anterior area, dorsally finely punctate, ventrally sparsely punctate (Fig. 4E); metapleuron with sparse irregular rugosities; notauli narrow, anteriorly punctate, fused posteriorly into rather flat punctate area; medio-posterior carina on 0.4 apical of the median lobe of mesoscutum (Fig. 4D); median and lateral lobes of mesoscutum finely punctate; scutellar sulcus wide and deep 0.5× as long as scutellum, with one median carina; scutellum finely punctate, as mesoscutum (Fig. 4D); propodeum without basal carina, with median closed areola, its surface mostly coriaceous (Fig. 4F).

***Wings***. Fore wing: pterostigma 2.6× as long as width medially (Fig. 4H); r: 3-SR: SR1 = 5: 11: 44; 2-SR: 3-SR: r-m: 2-M = 12: 11: 7: 19; vein r-m reclivous; m-cu far postfurcal; cu-a: 1-CU1: 2-CU1: 3-CU1 = 6: 6: 12: 6; vein 1-SR short; 1-M distinctly basally curved; vein 2A indicated by a weakly sclerotized base, medio-apically unsclerotized; vein a unsclerotized (Fig. 4H). Hind wing: M+CU: 1-M: cu-a: 1r-m = 21: 5: 7: 7 (Fig. 4G); marginal cell of hind wing more or less evenly widening apicad and apically comparatively wide (Fig. 4G); vein cu-a gradually curved and area basad of it sparsely setose.

***Legs***. Length of fore tarsus 1.4 × fore tibia; length of hind tarsus 0.9× hind tibia; hind tarsal claw simple; length of femur, tibia and basitarsus of hind leg 3.3, 6.3, and 6.0× their maximum widths, respectively; length of inner and outer hind tibial spurs 0.30 and 0.27× hind basitarsus, respectively.

***Metasoma***. Length of first tergite 1.2× its posterior width, its surface largely smooth (Fig. 4J); dorsal carinae strong basally, extending up to 0.6 tergite; second tergite 1.1 × longer third tergite; second suture distinct; length of ovipositor sheath 1.3× fore wing; ovipositor straight.

***Colour***. Head black; propleuron yellow; mesonotum yellowish brown or pale brown; propodeum brown; first metasomal tergite brown and following tergites brownish yellow; all sternites brownish yellow.

**Male**. Unknown.

##### Etymology.

The new species is named after the type locality, Ha Tinh province, North-Central Vietnam.

##### Biology.

Unknown.

##### Distribution.

North-Central Vietnam: Ha Tinh province.

#### Ussurohelcon mellicentralis

Taxon classificationAnimaliaHymenopteraBraconidae

Long sp. nov.

C2067955-EEB8-5472-9B04-52F6E5D7C5CD

https://zoobank.org/E068E0CD-7147-4F41-A386-6951617B95C9

Figs 5, 6, 7

##### Type material.

***Holotype***: • ♀, labelled “Hel.**072**” (IEBR), North-Central Vietnam: Ha Tinh, Huong Son, forest, 18°22'N, 106°13'E, 900 m, April 20–28, 1998, Malaise [trap], AMNH, K Long. ***Paratype***: • 1♂, labelled “Hel.**074**”, (IEBR), ibid. but May 5, 1998, Malaise [trap], AMNH, K Long.

##### Notes.

*Ussurohelcon mellicentralis* sp. nov. is the most similar to *U. hatinh* sp. nov., but it differs from the latter by the following characters: 1) occipital carina medio-dorsally angularly interrupted (vs complete in *U. hatinh*); 2) vein 3-SR of fore wing distinctly shorter than 2-SR (vs equal in length in *U. hatinh*); and 3) mesonotum yellow (vs yellowish brown in *U. hatinh*). Differences between *U. mellicentralis* sp. nov. and *U. mocchau* sp. nov. are indicated in the key.

**Figure 5.**
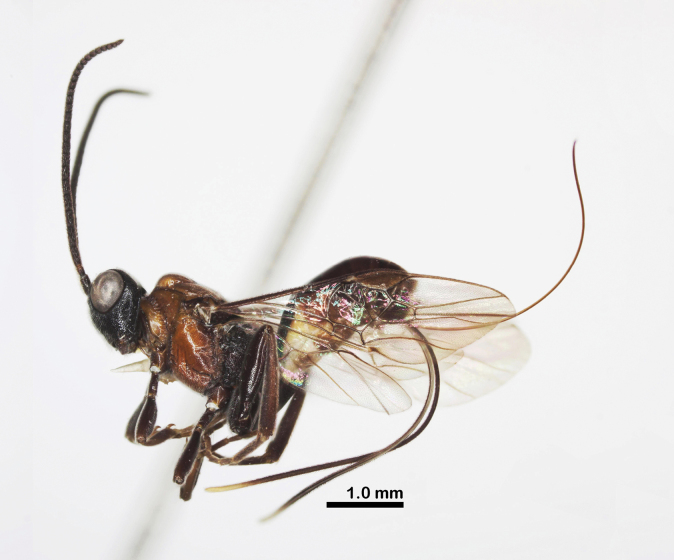
Habitus of *Ussurohelcon mellicentralis* Long, sp. nov., holotype, female, lateral view.

##### Description.

Holotype, ♀, length of body 5.2 mm, fore wing 4.7 mm, ovipositor sheath 4.7 mm (Fig. 5).

**Figure 6.**
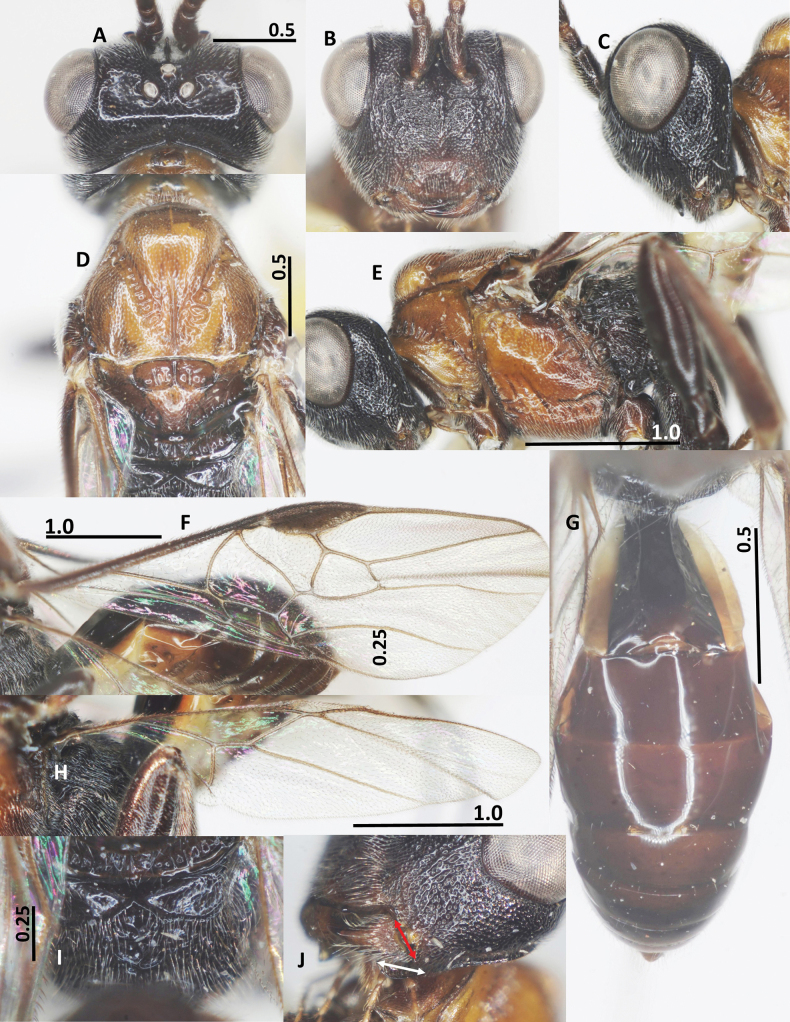
*Ussurohelcon mellicentralis* Long, sp. nov., holotype, female. **A**. Head, dorsal view; **B**. Head, frontal view; **C**. Head, lateral view; **D**. Mesonotum, dorsal view; **E**. Mesosoma, lateral view; **F**. Fore wing; **G**. Metasoma, dorsal view; **H**. Hind wing; **I**. Propodeum; **J**. Occipital flange and mandible, red arrow indicates width of mandible, and white arrow indicates length of ventral occipital flange. Scale bars in mm.

***Head***. Antenna with 26 flagellomeres; antenna of ♀ without white band; scapus 2.6× its maximum width; length of first flagellomere 1.1× second one; length of first, second and penultimate flagellomeres 2.3, 2.2, and 1.0× their width, respectively; scapus ventrally densely setose, length of scapus 2.1× its maximum width; length of maxillary palp 0.4× height of head; in dorsal view, length of eye 2.1× temple (Fig. 6A); in dorsal view, occipital carina complete, mediodorsally nearly angulated; ocelli small, OOL: OD: POL = 13: 4: 6; frons weakly depressed, medially smooth, laterally rugo-punctate; vertex and temple finely punctate (Fig. 6A); lamella acute between antennal sockets, which protrudes above depression then fused into median ridge below antennal sockets; in frontal view, width of face 1.4× length of face and clypeus medially combined; eye 1.4× as long as malar space; malar space 1.8× basal width of mandible (Fig. 6J); mandible basally rugose, apically smooth; face antero-laterally largely rugose, medially rugo-punctate, area between antennal sockets transversely rugulose (Fig. 6B); clypeus without median depression, finely rugo-punctate; ventral margin of clypeus with more or less pointed medial process; occipital flange protruding behind mandible 1.25× basal width of mandible (Fig. 6J); in lateral view, eye 1.1× and 1.1× and as long as its width, and malar space, respectively; malar space largely rugo-punctate (Fig. 6J); mandible basally rugo-punctate, apically smooth, ventrally with sparse, long setae.

***Mesosoma***. In lateral view mesoscutum rather flat posteriorly; length of mesosoma 1.4× its height (Fig. 6E); pronotal side densely crenulate medially, coriaceous ventrally, finely punctate dorsally (Fig. 6E); propleuron narrow, finely punctulate; prepectal carina complete, strong; postpectal carina present only medio-posteriorly; precoxal sulcus wide, situated low, shallower impressed anteriorly, deeper posteriorly, coriaceous to nearly smooth; mesopleuron mostly with sparse fine punctures (Fig. 6E); metapleuron largely rugulose; notauli rather wide, sparsely crenulate, fused into depressed coriaceous area posteriorly; medio-posterior carina on 0.5 apical of the median lobe of mesoscutum (Fig. 6D); middle lobe of mesoscutum convex antero-medially; median and lateral lobes of mesoscutum finely punctulate; scutellar sulcus wide and deep 0.5× as long as scutellum, with one median carina and two smaller lateral carinae (Fig. 6D); scutellum convex and nearly smooth; propodeum without basal carina, with median closed areola, its surface with sparse transverse rugae (Fig. 6I).

**Figure 7.**
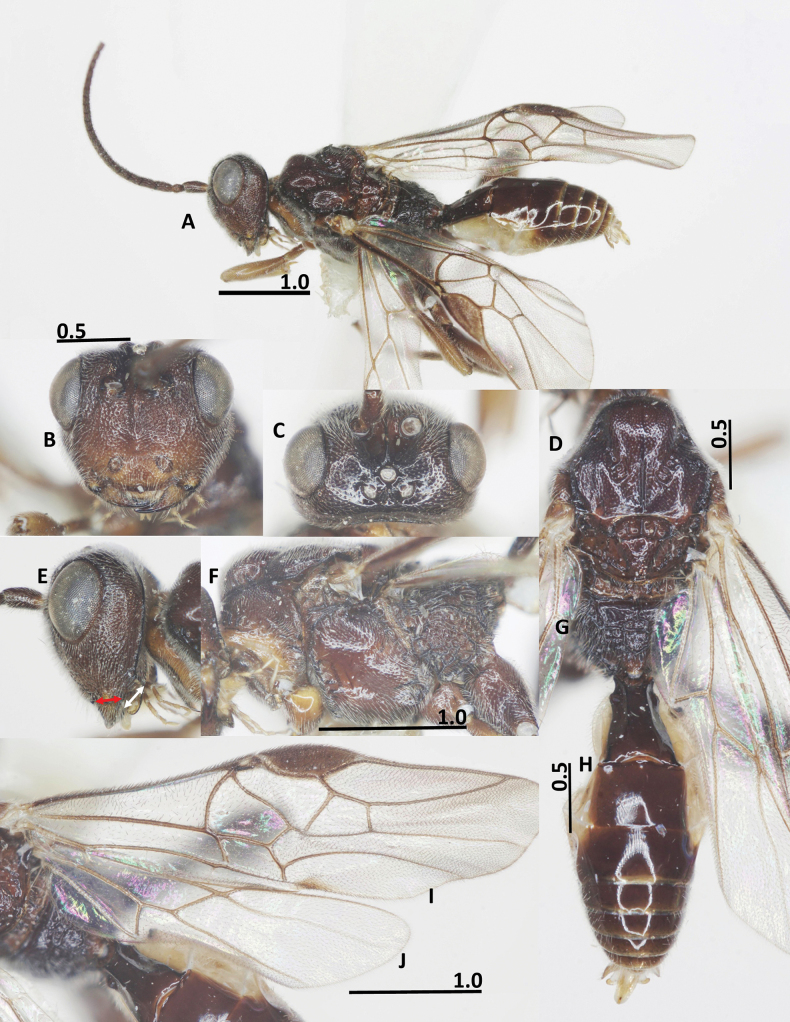
*Ussurohelcon mellicentralis* Long, sp. nov., paratype, male. **A**. Habitus, dorso-lateral view; **B**. Head, frontal view; **C**. Head, dorsal view; **D**. Mesonotum, dorsal; **E**. Occipital flange and mandible, red arrow indicates width of mandible, and white arrow indicates length of ventral occipital flange; **F**. Mesosoma, lateral view; **G**. Propodeum; **H**. Metasoma, dorsal view; **I**. Fore wing; **J**. Hind wing. Scale bars in mm.

***Wings***. Fore wing: pterostigma 2.6× width medially (Fig. 6F); r: 3-SR: SR1 = 7: 14: 62; 2-SR: 3-SR: r-m: 2-M = 16: 14: 10: 25; vein r-m reclivous; m-cu far postfurcal; cu-a: 1-CU1: 2-CU1 = 8: 6: 18; vein 1-SR short; 1-M distinctly curved; no trace of veins 1A and 2A (Fig. 6F). Hind wing: M+CU: 1-M: cu-a: 1r-m = 42: 10: 13: 17 (Fig. 6H); vein cu-a slightly curved and area basad of it sparsely setose (Fig. 6H).

***Legs***. Length of fore tarsus as long as fore tibia; tarsal claw simple, large; length of femur, tibia and basitarsus of hind leg 3.2, 6.6 and 5.3× their width, respectively; length of inner and outer hind tibial spurs 0.30 and 0.25× hind basitarsus, respectively.

***Metasoma***. Length of first metasomal tergite 1.3× its posterior width, its surface largely smooth (Fig. 6G), and dorso-lateral carinae basally strong, up to basal 0.6 of tergite; second tergite as long as third tergite, second suture distinct; length of ovipositor sheath as long as fore wing; ovipositor curved upward.

***Colour***. Head dark brown; antenna brown; glossa and palpi yellowish brown; mesonotum yellow; propodeum dark brown; fore and middle legs yellow, except fore and middle femur brown; hind leg dark brown, except tibia and tarsus pale brown; wing veins brownish yellow; first metasomal tergite brown; the following tergites brownish yellow; first sternite whitish yellow and following sternites yellow; ovipositor sheath brown, yellow apically; ovipositor yellow.

**Male**. (Fig. 7A), labelled “Hel.**074**”: antenna incomplete, with 19 flagellomeres remaining; length of body 4.7 mm, fore wing 3.8 mm; antenna incomplete, with 19 flagellomeres remaining; length of scapus 2.4× its maximum width; occipital flange protruding behind mandible as long as basal width of mandible (Fig. 7E); in dorsal view, OOL: OD: POL = 12: 4: 7; frons weakly depressed, smooth medially, rugo-punctate laterally; vertex and temple finely punctate (Fig. 7C); length of pterostigma 2.7 × its width medially; 1-CU1: cu-a: 2-CU1: 3-CU1 = 5: 7: 16: 7; r: 2-SR: 3: SR: r-m: SR1 = 5: 17: 9: 7: 37 (Fig. 7I); hind wing: M+CU: 1-M: cu-a: 1r-m = 40: 10: 11: 13 (Fig. 7J); vein cu-a slightly curved and area basad of it sparsely setose (Fig. 7J); notauli rather wide, sparsely crenulate, divided by median carina posteriorly; medio-posterior carina on 0.5 apical of the median lobe of mesoscutum; median and lateral lobes of mesoscutum finely punctulate; scutellar sulcus wide and deep, 0.6× as long as scutellum, with three medial carinae (Fig. 7D); scutellum convex and nearly smooth; propodeum without basal carina (Fig. 7G); length of first metasomal tergite 1.5× its posterior width (Fig. 7H); dorso-lateral carinae on 0.7 basal of tergite; surface of tergite largely smooth.

***Colour***. Head brownish yellow (dark brown in female); antenna pale brown; palpi yellow; propleuron and pronotal side ventrally yellow; mesonotum brownish yellow (propodeum dark brown in female); fore and middle legs yellow; hind leg brownish yellow, except basitarsomere and second to third tarsomeres brown. Head brownish yellow (dark brown in female); mesonotum brownish yellow (mesonotum yellow in female contrasting to dark brown propodeum).

##### Etymology.

“Mel, mellis” is Latin for “honey”, and “centrum”, in reference to the yellow mesonotum.

##### Biology.

Unknown.

##### Distribution.

North-Central Vietnam (Ha Tinh province).

#### Ussurohelcon mocchau

Taxon classificationAnimaliaHymenopteraBraconidae

Long & van Achterberg sp. nov.

931953BF-D52B-5198-863B-52B0EA2B84A1

https://zoobank.org/60798EB7-8873-4740-BEEF-8FEFF474F75A

Figs 8, 9

##### Type material.

***Holotype***: • ♀, labelled “Hel.**112**” (IEBR), Northwest Vietnam: Son La, Moc Chau, Tan Lap commune, forest, wood falls, 20°57'6"N, 104°37'31"E, 650 m, sweep (net), 5.vi.2024, KD Long. ***Paratypes***: • 11 ♀, data as in holotype: 7 ♀ (IEBR), labelled “Hel.**111**”, “Hel.**113**”, “Hel.**123**”, “Hel.**124**”, “Hel.**125**”, “Hel.**126**”, “Hel.**127**”; 2♀ (DThU), labelled “Hel.**115**”, “Hel.**116**”; 1♀ (AMNH), labelled “Hel.**117**”; 1♀ (RMNH), labelled “Hel.**118**”.

##### Notes.

*Ussurohelcon mocchau* sp. nov. shares with other species from Vietnam a frons with a rather deep median depression and an inclivous vein r-m, but it differs from the others in having the following characters: face with median longitudinal groove from frons to clypeus (vs without longitudinal groove); vein 1-SR of fore wing absent (vs 1-SR of fore wing present and short); and vein 1-M weakly curved (vs vein 1-M of fore wing basally or medially distinctly curved).

##### Description.

**Figure 8.**
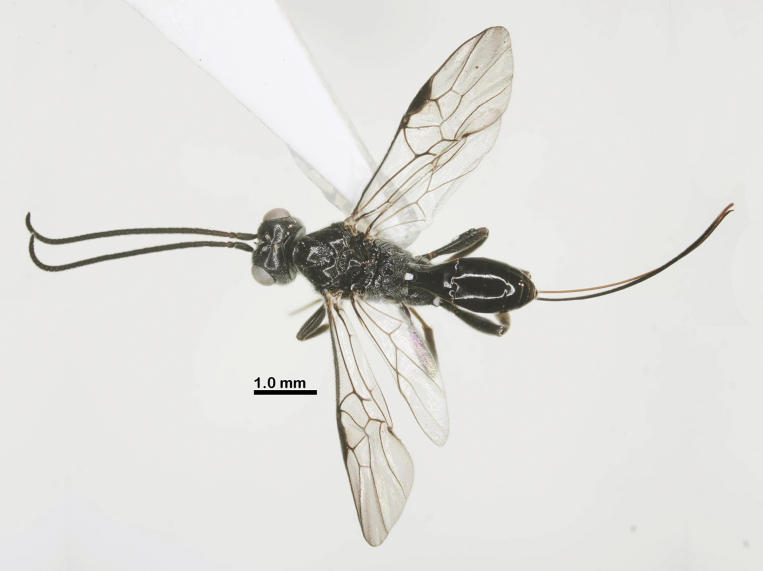
Habitus of *Ussurohelcon mocchau* Long & van Achterberg, sp. nov., holotype, female, dorsal view.

Holotype, ♀, length of body 5.2 mm, fore wing 4.5 mm, ovipositor sheath 4.0 mm (Fig. 8).

**Figure 9.**
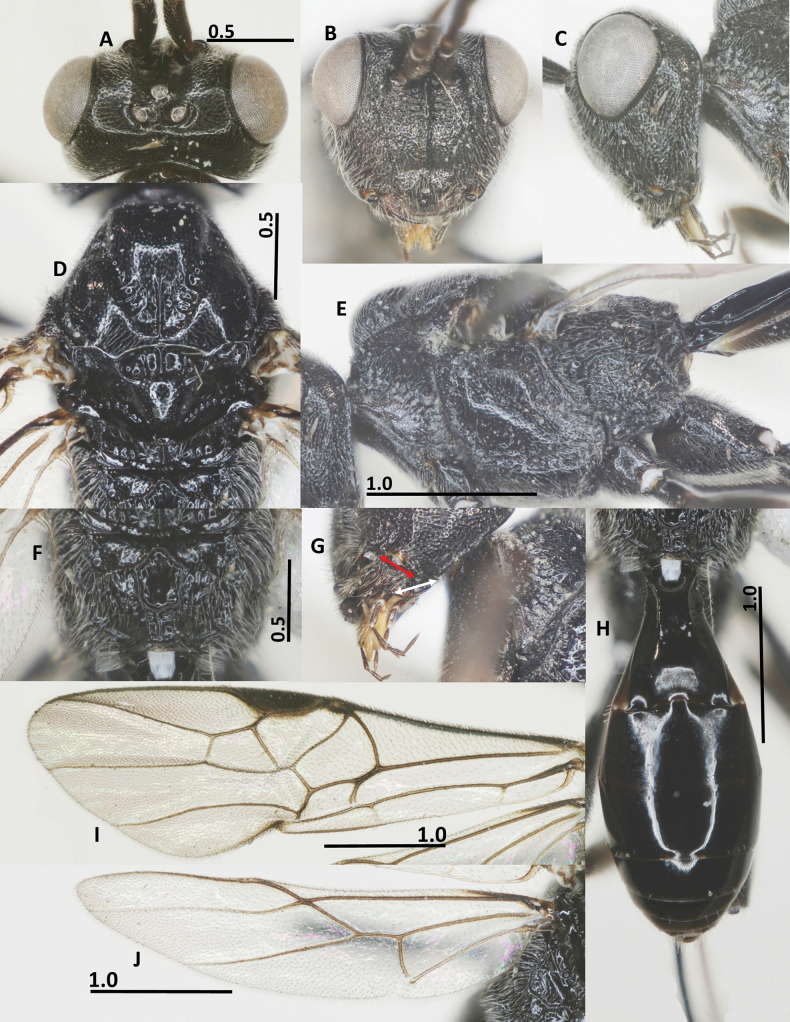
*Ussurohelcon mocchau* Long & van Achterberg, sp. nov., holotype, female. **A**. Head, dorsal view; **B**. Head, frontal view; **C**. Head, lateral view; **D**. Mesonotum, dorsal view; **E**. Mesosoma, lateral view; **F**. Propodeum; **G**. Occipital flange and mandible, red arrow indicates width of mandible, and white arrow indicates length of ventral occipital flange; **H**. Metasoma, dorsal view; **I**. Fore wing; **J**. Hind wing. Scale bars in mm.

***Head***. Antenna with 27 flagellomeres; antenna without white band; length of scapus 2.6× its maximum width; length of first flagellomere 1.1× second one; length of first, second and penultimate flagellomeres 3.0, 2.75, and 1.0× their width, respectively; in dorsal view, occipital carina complete, rather deeply curved (Fig. 9A); head 2.05× as wide as length medially; length of eye 2.5× temple (Fig. 9A); in dorsal view, occipital carina complete, medio-dorsally angulated; ocelli small-sided, OOL: OD: POL = 12: 5: 6 (Fig. 9A); frons smooth anteriorly, laterally finely rugo-punctate (Fig. 9A); vertex and temple sparsely punctate; in frontal view, glossa rather long, bilobed (Fig. 9B); length of maxillary palp 0.4× height of head; width of face 1.5× length of face and clypeus medially combined; eye length 1.3× malar space; lamella blunt, protruding above median depression of frons; face with an obtuse ridge below antennal sockets, fused into median longitudinal groove extending to clypeus (Fig. 9B); face largely rugo-punctate; clypeus weakly depressed; malar space with discrete punctures (Fig. 9C, G); clypeus rugo-punctate; ventral margin of clypeus with distinct pointed medial process; in lateral view, flange wide, protruding behind mandible 1.1× as long as basal width of mandible (Fig. 9G); eye 1.4 and 2.2× as long as wide, and temple medially, respectively; malar space 3.0× as long as basal width of mandible; mandibles basally rugo-punctate, apically smooth (Fig. 9G).

***Mesosoma***. In lateral view, mesoscutum raised posteriorly; length of mesosoma 1.5× its height (Fig. 9E); pronotal side largely crenulate medially, finely punctate ventrally and dorsally (Fig. 9E); propleuron finely punctulate; prepectal carina complete, strong; postpectal carina present only medio-posteriorly; precoxal sulcus wide, shallowly impressed throughout, rugulose; remainder of mesopleuron finely punctate (Fig. 9E); metapleuron largely rugose; notauli rather deep anteriorly, posteriorly widely crenulate; medio-posterior carina on 0.4 apical of the median lobe of mesoscutum (Fig. 9D); median and lateral lobes of mesoscutum finely punctulate; scutellar sulcus deep and wide 0.6× as long as scutellum, with 3 carinae; scutellum almost smooth, with sparse fine punctures (Fig. 9D); propodeum without basal carina, with median closed areola, connecting carina apically; propodeum laterally densely setose, surface of areola coriaceous (Fig. 9F).

***Wings***. Fore wing: pterostigma 2.8× as long as width medially (Fig. 9I); r: 3-SR: SR1 = 6: 15: 56; 2-SR: 3-SR: r-m = 16: 15: 10; 3-SR: 2-M = 15: 24; vein r-m reclivous; m-cu far postfurcal; cu-a: 1-CU1: 2-CU1: 3-CU1 = 8: 8: 16: 9; vein 1-SR absent; 1-M distinctly curved; veins a and 2A weakly sclerotized (Fig. 9I). Hind wing: M+CU: 1-M: cu-a: 1r-m = 33: 10: 12: 13 (Fig. 9J); vein cu-a nearly perpendicular, weakly curved and area basad of it setose (Fig. 9J).

***Legs***. Length of fore tarsus 1.2× fore tibia; hind tarsal claw simple, large; length of femur, tibia and basitarsus of hind leg 3.90, 6.50 and 5.25× their width, respectively; length of inner and outer hind tibial spurs 0.30 and 0.25× hind basitarsus, respectively.

***Metasoma***. Length of first metasomal tergite 1.0× its posterior width, its surface largely smooth (Fig. 9H), and dorso-lateral carinae strong basally, extending up to 0.5 basal of the tergite; second tergite 1.1× longer third tergite; second suture distinct; length of ovipositor sheath 0.9× fore wing; ovipositor curved upward.

***Colour***. Black; antenna black; palpi dark brown in contrast to yellow bilobed glossa (Fig. 9B); all legs black, except tarsus pale brown; wing veins brown; ovipositor sheath dark brown, yellow apically; ovipositor yellow.

**Male**. Unknown.

##### Etymology.

The new species is named after the locality, Moc Chau district (Son La province) Northwestern Vietnam, where the holotype was collected.

##### Biology.

**Figure 10.**
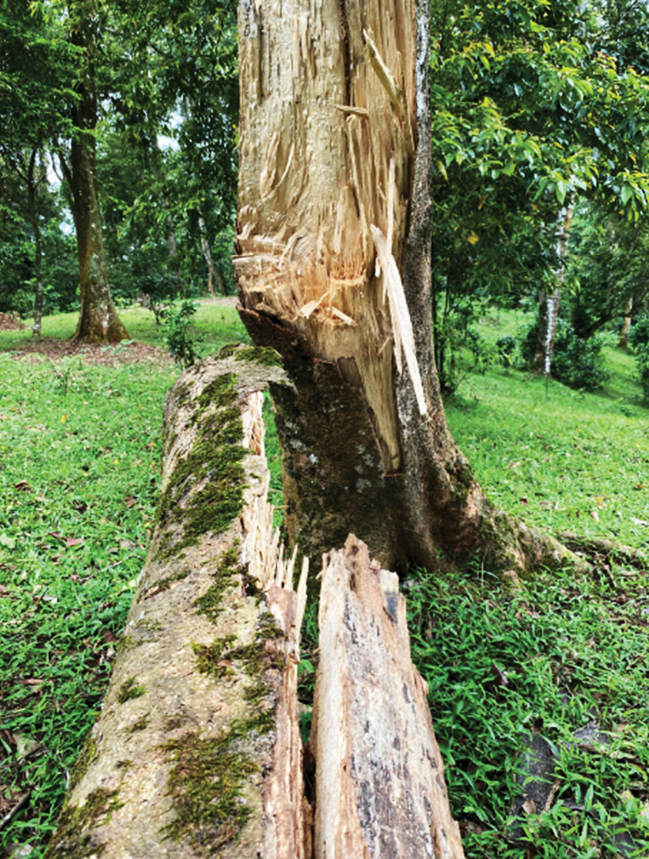
Habitat of *Ussurohelcon mocchau* Long & van Achterberg, sp. nov.

Unknown, but a series of females were collected on dead fallen wood (Fig. 10) suggesting that they may be parasitoids of coleopterous larvae.

##### Distribution.

Northwestern Vietnam: Son La province.

#### Ussurohelcon similis

Taxon classificationAnimaliaHymenopteraBraconidae

Long sp. nov.

A66548F0-5D4D-5C1C-9D1D-12CF274C000A

https://zoobank.org/1AB22F15-A253-4E83-AFB4-2F48D30BE7EE

Figs 11, 12

##### Type material.

***Holotype***: • ♀, labelled “Hel.**024**” (IEBR), North-Central Vietnam: Ha Tinh, Huong Son, forest, 18°22'N, 106°13'E, 900 m, May 5, 1998, Malaise [trap], AMNH, K. Long.

##### Notes.

*Ussurohelcon similis* sp. nov. is similar to *U. nigricornis* van Achterberg from Oriental (Malaysia) but differs by the following characters: 1) occipital flange short and narrow (vs wide in *U. nigricornis*); 2) vein r of fore wing 0.35× length of 2-SR (vs 0.5× in *U. nigricornis*); and 3) length of first metasomal tergite 1.75× its posterior width (vs 1.4× in *U. nigricornis*).

##### Description.

**Figure 11.**
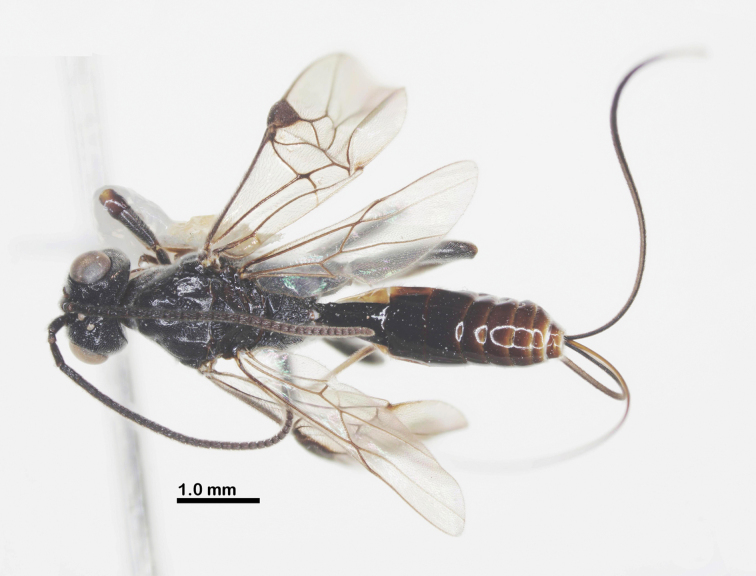
Habitus of *Ussurohelcon similis* Long, sp. nov., holotype, female, dorsal view.

Holotype, ♀, length of body 7.5 mm, fore wing 5.5 mm, ovipositor sheath 6.5 mm (Fig. 11).

**Figure 12.**
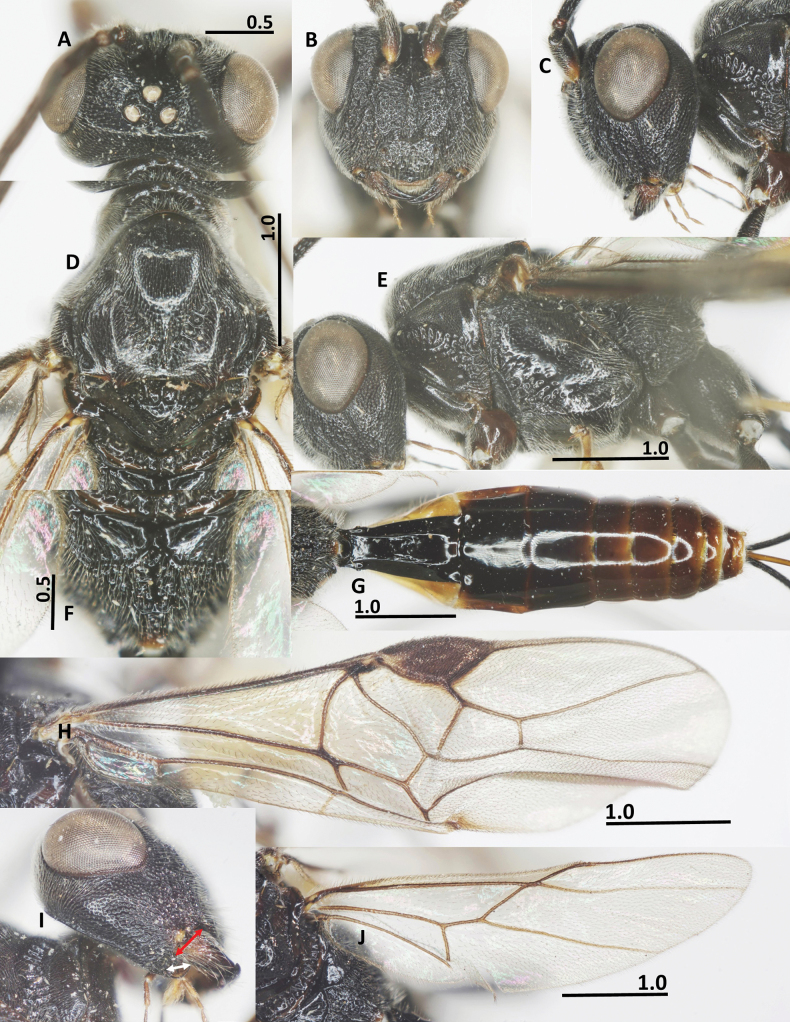
*Ussurohelcon similis* Long, sp. nov., holotype, female. **A**. Head, dorsal view; **B**. Head, frontal view; **C**. Head, lateral; **D**. Mesonotum, dorsal view; **E**. Mesosoma, lateral view; **F**. Propodeum; **G**. Metasoma, dorsal view; **H**. Fore wing; **I**. Occipital flange and mandible, red arrow indicates width of mandible, and white arrow indicates length of ventral occipital flange; **J**. Hind wing. Scale bars in mm.

***Head***. Antena with 27 flagellomeres; antenna of ♀ without white band; length of scapus 2.4× its maximum width; length of first flagellomere 1.1× second one; length of first, second, and penultimate flagellomeres 3.4, 3.2, and 1.0× their widths, respectively; in dorsal view, length of maxillary palp 0.4× height of head; occipital carina dorsally complete, weakly angularly curved; head 2.0× as wide as length medially; eye 2.5× as long as temple (Fig. 12A); frons anteriorly smooth, laterally rugo-punctate; vertex and temple punctate; occipital carina dorsally complete, medio-dorsally roundly curved; ocelli small, OOL: OD: POL = 10: 4: 5 (Fig. 12A); in frontal view, glossa short; between antennal sockets lamella blunt, protruding above depression; width of face 0.8× length of face and clypeus combined; eye length 1.2× malar space; malar space 2.6× mandible width; face with obtuse median ridge, largely rugo-punctate, area close to antennal sockets with few transverse rugosities (Fig. 12B); clypeus without depression, largely rugo-punctate as face (Fig. 12B); ventral margin of clypeus with obtuse medial process; occipital flange wide, protruding behind mandible, 0.8× as long as basal width of mandible (Fig. 12C, I); in lateral view, eye length 1.35× its width and 2.3× (as long as and temple medially; malar space 2.6× as long as basal width of mandible; mandible basally rugo-punctate, apically smooth, ventrally with long setae (Fig. 12I).

***Mesosoma***. In lateral view, mesoscutum posteriorly flat; length of mesosoma 1.55× its height (Fig. 12E); pronotal side medio-anteriorly crenulate, medio-posteriorly reticulate-rugulose, dorsally and ventrally coriaceous (Fig. 12E); propleuron fattened, coriaceous; prepectal carina complete, strong; postpectal carina present only medio-posteriorly; precoxal sulcus wide, anteriorly shallowly impressed, posteriorly deeper; precoxal sulcus anteriorly largely rugulose, but deeper near postpectal carina, posteriorly nearly coriaceous; remainder of mesopleuron shiny, with sparse, fine punctures (Fig. 12E); metapleuron areolate-rugose; anteriorly notauli deep and narrower, crenulate, posteriorly widely crenulate (Fig. 12E); mesoscutal lobes with sparse fine punctures (Fig. 12D); medio-posterior carina on 0.4 apical of the median lobe of mesoscutum; scutellar sulcus wide and deep, 0.55× as long as scutellum, with five carinae; scutellum convex, finely and sparsely punctulate (Fig. 12D); propodeum with short basal carina, with median closed areola connecting transverse rugae antero-laterally and rugosity posteriorly; baso-lateral areas of propodeum smooth; areola setose and rugulose (Fig. 12F).

***Wings***. Fore wing: pterostigma 2.5× as long as width medially (Fig. 12H); vein 1-SR absent; r: 3-SR: SR1 = 5: 14: 44; 2-SR: 3-SR: r-m: 2-M = 13: 14: 6: 20; vein r-m subvertical; m-cu far postfurcal; cu-a: 1-CU1: 2-CU1: 3-CU1= 7: 4: 16: 7 ; 1-M distinctly curved basally; veins 1A and 2A developed, unsclerotized (Fig. 12H). Hind wing: M+CU: 1-M: cu-a: 1r-m = 35: 10: 8: 12 (Fig. 12J); vein cu-a weakly curved and area basad of it sparsely setose (Fig. 12J).

***Legs***. Length of fore tarsus 1.2× fore tibia; hind tarsal claw simple, large; length of femur, tibia and basitarsus of hind leg 4.00, 6.90 and 5.75× their maximum width, respectively; length of inner and outer hind tibial spurs 0.3 and 0.2× hind basitarsus, respectively.

***Metasoma***. Length of first tergite 1.75× its posterior width, its surface largely smooth (Fig. 12G), and dorsal carinae strong, extending on 0.8 basal of the tergite; second tergite 1.1× longer third tergite; second suture indistinct medially (Fig. 12G); length of visible ovipositor sheath 1.2× fore wing.

***Colour***. Black; antenna dark brown, except scapus basally yellow; palpi basally brown, apically yellow; fore leg brown, except apical femur, tibia, and tarsus yellow; middle leg brown, except tibia and tarsus yellow; hind leg brown, except coxa black; wing veins brown; wing membrane hyaline; first metasomal tergite blackish brown; second and third tergites brown; the following tergites brownish yellow; sternites whitish yellow; ovipositor sheath brown, apically yellow; ovipositor yellow.

**Male**. Unknown.

##### Etymology.

Named from “simulo” (Latin for “imitate, copy”), because this new species is similar to *W. nigricornis* van Achterberg, 1994.

##### Biology.

Unknown.

##### Distribution.

North-Central Vietnam: Ha Tinh province.

#### Ussurohelcon tuyenquang

Taxon classificationAnimaliaHymenopteraBraconidae

Long sp. nov.

F764DF3A-925E-5B3C-9E6B-8685EC994950

https://zoobank.org/70486BC9-37C8-4C0D-B378-9358672383CF

Figs 13, 14

##### Type material.

***Holotype***: • ♀, labelled “Hel.**026**” (IEBR), Northeast Vietnam: Tuyen Quang, Na Hang NP, Son Phu, forest, Malaise [trap], 22°17'32"N, 105°28'19"E, 573 m, 25.viii.2017, KD Long.

##### Notes.

*Ussurohelcon tuyenquang* sp. nov. shares with other species with 1-SR of fore wing short and vein r-m of fore wing inclivous but can be distinguished by the following characters: first metasomal tergite 1.5× its apical width (vs 1.2–1.3× in the others); ovipositor sheath 1.5× fore wing (vs 1.0–1.3 in the others); and propleuron black, and mesonotum black (vs propleuron yellow or pale brown, and mesonotum entirely yellow or yellowish brown in the others).

##### Description.

**Figure 13.**
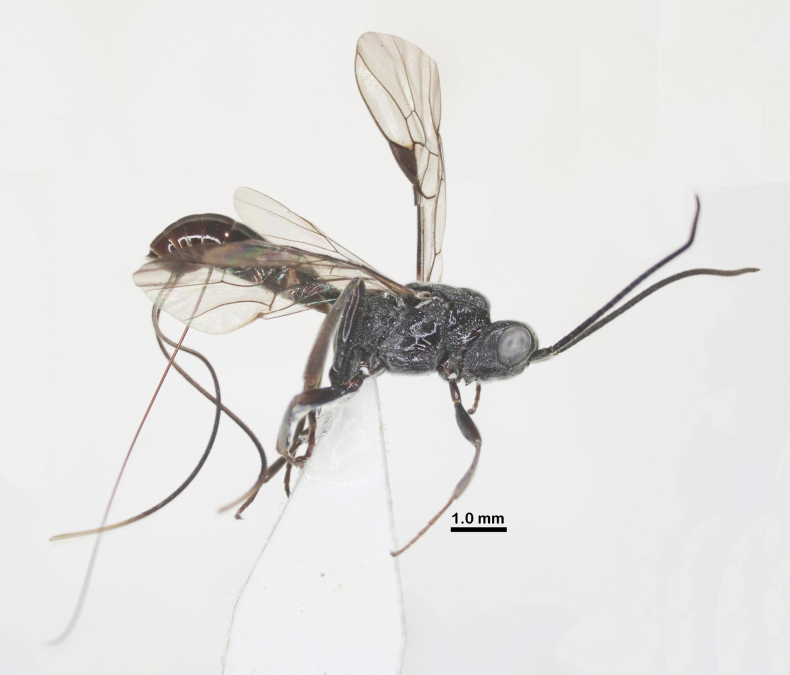
Habitus of *Ussurohelcon tuyenquang* Long, sp. nov., holotype, female, lateral view.

Holotype, ♀, length of body 8.0 mm, fore wing 5.8 mm, ovipositor sheath 8.5 mm (Fig. 13).

**Figure 14.**
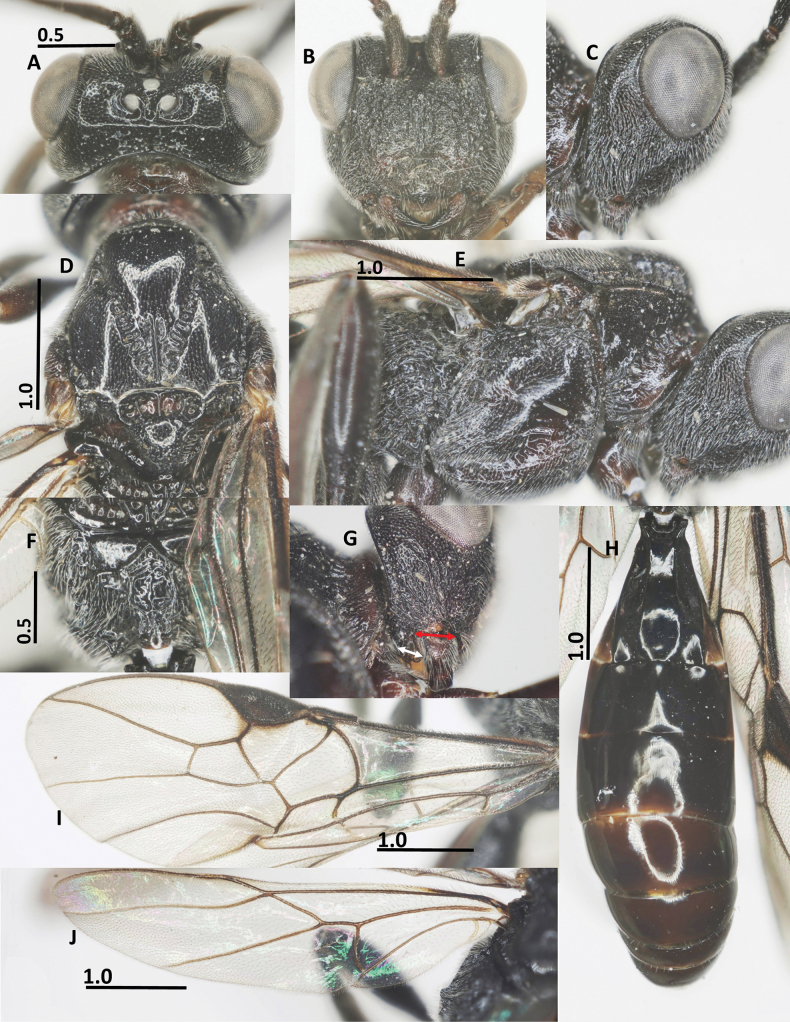
*Ussurohelcon tuyenquang* Long, sp. nov., holotype, female. **A**. Head, dorsal view; **B**. Head, frontal view; **C**. Head, lateral view; **D**. Mesonotum, dorsal view; **E**. Mesosoma, lateral view; **F**. Propodeum; **G**. Occipital flange and mandible, red arrow indicates width of mandible, and white arrow indicates length of ventral occipital flange; **H**. Metasoma, dorsal view; **I**. Fore wing; **J**. Hind wing. Scale bars in mm.

***Head***. Antena with 29 flagellomeres; antenna of ♀ without white band; length of scapus 2.6× its maximum width scapus; length of first flagellomere 1.1× second one; length of first, second, and penultimate flagellomeres 3.4, 3.0, and 1.0× their widths, respectively; in dorsal view, occipital carina complete, evenly medio-dorsally curved (Fig. 14A); frons medially depressed, laterally punctate; between antennal sockets lamella obtuse, protruding above depression, connecting ridge in face; head 2.2× as wide as length medially; length of eye 2.9× temple (Fig. 14A); ocelli small, OOL: OD: POL = 11: 4: 4 (Fig. 14A); vertex and temple with dense, fine punctures (Fig. 14A); in frontal view, glossa and palpi very short; clypeus with wide, triangular medio-posterior depression, ventral margin of clypeus with distinct pointed medial process; clypeus rugo-punctate; width of face 0.8× length of face and clypeus medially combined; eye 1.2× as long as malar space; malar space 2.1× basal width of mandible; face with obtuse median ridge, largely rugose (Fig. 14B); occipital flange wide protruding behind mandible, as long as basal width of mandible (Fig. 14G); in lateral view, eye length 1.7× as long as its width, and 2.6× malar space; malar space largely rugo-punctate; mandible twisted, with long and dense setae, basally rugo-punctate, medio-apically smooth (Fig. 14C, G).

***Mesosoma***. In lateral view, mesoscutum posteriorly weakly convex; length of mesosoma 1.55× its height (Fig. 14E); pronotal side largely crenulate medially, dorsally and ventrally shiny, with dense, fine punctures; propleuron narrow, coriaceous (Fig. 14E); prepectal carina complete, strong; postpectal carina present only medio-posteriorly; precoxal sulcus wide and shallowly impressed, but deeper near postpectal carina, weakly crenulate; remainder of mesopleuron (dorsally and ventrally) finely punctate (Fig. 4E); metapleuron reticulate-rugose; notauli rather wide and deep, extending close to scutellar sulcus, posteriorly widely crenulate (Fig. 14E); mesoscutal lobes with dense, fine punctures; medio-posterior carina on 0.4 apical of the median lobe of mesoscutum (Fig. 14D); scutellar sulcus deep and wide, 0.5× as long as scutellum, with three carinae; scutellum convex and finely punctate (Fig. 14D); propodeum laterally and posteriorly densely setose, with median closed areola, connecting transverse rugae antero-laterally and two posteriorly rugosities; baso-lateral areas of propodeum smooth; areola with sparse, irregular rugosities (Fig. 14 F).

***Wings***. Fore wing: pterostigma 2.6× as long as width medially (Fig. 14I); r: 2-SR: 3-SR: r-m: SR1 = 5: 12: 11: 7: 42; r: 3-SR: SR1 = 5: 11: 42; 2-SR: 3-SR: r-m = 12: 11: 7; 3-SR: 2-M = 11: 18; vein r-m reclivous; vein 1-M curved basally (Fig. 14I); cu-a far postfurcal; 1-CU1: cu-a: 2-CU1: 3-CU1 = 5: 7: 14: 7; vein 1-SR very short; 1-M distinctly curved throughout; vein r-m inclivous; vein 2A developed, sclerotized; vein a basally sclerotized; medio-apically pigmented (Fig. 14I). Hind wing: M+CU: 1-M: cu-a: 1r-m = 40: 10: 14: 15 (Fig. 14J); marginal cell of hind wing more or less evenly widening apicad and apically comparatively wide; vein cu-a apically curved and area basad of it largely glabrous (Fig. 14J).

***Legs***. Length of fore tarsus 1.4× fore tibia; length of femur, tibia, and basitarsus of hind leg 3.5, 7.1, and 7.7× their maximum width, respectively; hind inner and outer tibial spurs 0.25 and 0.2× hind basitarsus, respectively.

***Metasoma***. Length of first tergite 1.5× its posterior width, its surface largely smooth (Fig. 14H), and dorsal carinae basally strong, up to 0.7 basal of the tergite; second tergite 0.9× third tergite, second suture distinct (Fig. 14H); length of ovipositor sheath 1.5× fore wing; ovipositor straight.

***Colour***. Black, pronotum yellow; antenna black; glossa yellow; palpi basally brown, apically yellow; fore and middle legs brown, except tibia and tarsus yellow; hind coxa and femur black; hind tibia and tarsus brown; wing veins and setae brown; wing membrane hyaline; first to third metasomal tergites blackish brown to black and following tergites yellowish brown; sternites dirty yellow; ovipositor sheath brown, apically yellow; ovipositor yellow.

**Male**. Unknown.

##### Etymology.

The new species is named after the type locality, Tuyen Quang province, Northeastern Vietnam.

##### Biology.

Unknown.

##### Distribution.

Northeastern Vietnam: Tuyen Quang province.

### Remarks

The genus *Ussurohelcon* is newly recorded as part of Vietnam’s braconid fauna, and this paper includes 11 species, with the addition of six newly described species. Females of Vietnamese *Ussurohelcon* species can be distinguished by the following comparative characters: sculpture of frons; ratio of occipital flange and basal width of mandible; in fore wing, vein 1-SR absent or present; vein r-m vertical/subvertical or inclivous; veins 1A well sclerotized or unsclerotized; suture between second and third terga distinct (most species) or indistinct (*U. hagiang* sp. nov. and *U. similis* sp. nov.). Apart from the above-mentioned morphological characters, the colour pattern of the mesosoma seems to be an important character for distinguishing among *Ussurohelcon* species. A series of females of *U. mocchau* sp. nov. were collected from fallen dead wood, which hints at this species likely being a parasitoid of wood- or bark-boring coleopteran larvae.

## Supplementary Material

XML Treatment for Ussurohelcon

XML Treatment for Ussurohelcon hagiang

XML Treatment for Ussurohelcon hatinh

XML Treatment for Ussurohelcon mellicentralis

XML Treatment for Ussurohelcon mocchau

XML Treatment for Ussurohelcon similis

XML Treatment for Ussurohelcon tuyenquang

## Additional information

### Use of AI

No use of AI was reported.

### Author ORCIDs

Nguyen Thi Oanh https://orcid.org/0000-0002-3265-0580

Nguyen Van Dzuong https://orcid.org/0000-0003-2362-0088

Khuat Dang Long https://orcid.org/0000-0002-9237-7344

Cornelis van Achterberg https://orcid.org/0000-0002-6495-4853

Nguyen Duc Hiep https://orcid.org/0000-0003-4328-9652

### Data availability

All of the data that support the findings of this study are available in the main text.
